# Analysis of association between MALAT1 haplotype and the severity of normal‐tension glaucoma (NTG)

**DOI:** 10.1111/jcmm.15906

**Published:** 2021-10-02

**Authors:** Jin‐liang Yue, Shu‐feng Zheng

**Affiliations:** ^1^ Ophthalmology Department Zhoukou Central Hospital Zhoukou China; ^2^ Ophthalmology Department Eye Hospital Traditional Chinese Medicine Hospital of Yulin Yulin China

**Keywords:** IL‐6, MALAT1, MI‐RNA, NTG

## Abstract

MALAT1, which is disorderly expressed in the growth, invasion, migration and cancer cell apoptosis, was shown to be associated with normal‐tension glaucoma (NTG), a type of optic neuropathy. The haplotype in MALAT1 affects its expression and is correlated with human diseases like normal‐tension glaucoma (NTG). However, the underlying detailed mechanism remains unclear. In this study, we aimed to analyse the association between MALAT1 haplotype and the severity of NTG in a molecular level. Quantitative real‐time PCR, ELISA and luciferase assays were performed to establish the underlying signalling pathways. RNFL thickness, RA and C/D ratio were calculated for NTG patients. Accordingly, GGGT haplotype was demonstrated to be associated with a decreased risk of NTG. The MALAT1 level in serum of NTG patients carrying GGGT haplotype was significantly decreased compared with NTG patients carrying other haplotypes, along with elevated miR‐1 expression and diminished IL‐6 expression. NTG patients carrying GGGT haplotype had thicker RNFL and RA, but a smaller C/D ratio. Sequence analysis found potential target sites of miR‐1 on MALAT1 and IL‐6, and luciferase assay confirmed the inhibitory effect of miR‐1 on MALAT1 and IL‐6 expression. Meanwhile, MALAT1 also down‐regulated miR‐1 expression and consequently up‐regulated IL‐6 expression. This study presented evidence for a regulatory network containing MALAT1, miR‐1 and IL‐6, and further demonstrated the effect of MALAT1 haplotype on the risk and severity of NTG.

## 1 INTRODUCTION

As a type of optic neuropathy, normal‐tension glaucoma (NTG) does not cause abnormal intraocular pressure (IOP) in its patients. Therefore, it is hard to distinguish NTG from glaucomatous optic neuropathy.^1^, ^2^ In addition, it is hard to estimate the impact of NTF on the thickness of central cornea during IOP measurement.^3^ Furthermore, few studies have compared the clinical features of different NTG.

As a type of stimulatory cytokines regulating immune reactions as well as inflammatory signalling, IL‐6 exerts its effects by activating the secretion of C‐reactive proteins.^4^ IL‐6 can also stimulate the hypothalamic‐pituitary‐adrenal signalling as well as regulating hematopoiesis.^5^ IL‐6 is mainly synthesized by monocytes, lymphocytes and adrenal cortex, although it is not synthesized in adrenal medulla.^6^ Single‐nucleotide polymorphism (SNP) in IL‐6 was demonstrated to be involved in glaucoma pathogenesis in patients with NTG. The effect of IL‐6 SNP on NTG may be caused by the close relationship between the level of IL‐6 and neurodegenerative disorders. SNPs in IL‐6 are implicated in Alzheimer's disease, sclerosis and myasthenia gravis.^7^ During glaucoma, the neurotoxicity of amyloid‐β induces the apoptosis of RGCs.^8^ Fisher et al suggested that IL‐6 exerts negative effects on the survival of neurons in the early stage of brain trauma.^9^ In addition, the SNPs in IL‐6 were implicated in a wide range of human disorders by altering the level of IL‐6 expression.^10^ SNPs in IL‐6 were also found to affect the functions of IL‐6. For example, a G/C SNP located in the promoter of IL‐6 can affect the serum expression of IL‐6 as well as the transcription of IL‐6 DNA. Fishman et al showed that the concentration of serum IL‐6 in subjects carrying the GG genotype of this SNP was twice that in subjects carrying the C allele of this SNP.^11^

Long non‐coding RNA, abbreviated as lncRNA, has been implicated in the onset of various malignancies.^12^, ^13^ In addition, dysregulated lncRNAs are involved in the EMT and metastasis of human cancers.^14^ LncRNAs also function as competing endogenous RNAs (ceRNAs) to sponge microRNAs (miRNAs).^15^, ^16^ An lncRNA termed MALAT1 contains about 8,000 nucleotides and was considered to be disorderly expressed in the growth, invasion, migration, as well as apoptosis of a wide range of different cancer cells, including lung cancer cells, gastric cancer cells, as well as liver cancer cells.^17^, ^18^, ^19^, ^20^, ^21^

Reduced expression of MALAT1 results in reduced visual activity as well as the apoptosis of retinal cells.^22^ Furthermore, MALAT1 promotes cell growth through the activation of PI3K/Akt signalling.^23^, ^24^ Nakazawa et al produced an animal model of optic nerve damage to show that the activation of PI3K/Akt signalling generates neuroprotective effects against optic nerve damage as well as retinal damage.^22^ Furthermore, P13K/Akt cascade aids the growth of RGCs.^25^ These studies showed that MALAT1 reduces the apoptosis of RGCs during glaucoma via enhancing the activity of the P13K/Akt cascade.^26^

All above data suggested that the knockdown of MALAT1 reduces the growth as well as the migration of neuron cells while accelerating the apoptosis of these cells by elevating the expression of miR‐1. Chou et al showed that MALAT1 helped the migration as well as the invasion of tumour cells by working as a miR‐1 sponge in patients with breast cancer.^27^ Jin et al showed that the silencing of MALAT1 reduced the growth as well as migration of tumour cells while promoting the apoptosis of these cells via the miR‐1/slug signalling in breast cancer patients.^28^

It has been reported that the haplotype in MALAT1 was associated with many human diseases by alternating MALAT1 expression.^29^ It was also reported that miR‐1 may reciprocally interact with MALAT1 by functioning as a competing endogenous RNA of MALAT1, while IL‐6 is a possible target of miR‐1. Furthermore, IL‐6 has been reported to be associated with the risk and severity of glaucoma.^30^, ^31^ In this study, we collected blood samples from subjects with and without normal‐tension glaucoma (NTG) and studied the association between the haplotype in MALAT1 and the risk and severity of NTG.

## 2 MATERIALS AND METHODS

### 2.1 Human subjects sample collection

A total of 827 participants were enrolled in our research, which consisted of 402 participants diagnosed with NTG (the NTG group, N = 402) and 425 participants free of any health problems (the Control group, N = 425). The peripheral blood samples were collected from above subjects and the information of these participants, including their age, sex, history of systemic diseases such as hypertension, diabetes mellitus and hyperlipidaemia, and body mass index were collected.

In addition, the 402 NTG patients were divided into 6 groups according to their haplotypes of MALAT1 (CACC, GGGT, CGCC, CAGC, GAGT and CACT). Then, the serum samples of all NTG patients with different haplotypes of MALAT1 were collected to evaluate their expression of MALAT1, miR‐1 and IL‐6.

### 2.2 C/D ratio

The C/D ratio in the NTG patients of all 6 groups was estimated independently by 2 specialists with rich experience in the diagnosis and treatment of glaucoma. The calculation of C/D ratio was done using slit‐lamp ophthalmoscopy in conjunction with the examination of head of the optic nerve in each patient. Based on the morphological change observed in the optic nerve disc in each patient, the 402 NTG patients of this study were further classified into two different groups. In group 1, the C/D ratio of the NTG patients was in a range of 0.3 to 0.7, which indicated the early to medium stage in the development and progression of NTG. In group 2, the C/D ratio of the NTG patients was in a range of 0.7 to 1.0, which indicated the advanced stage in the development and progression of NTG.

### 2.3 Genotyping by TaqMan assay

The genomic DNA in collected serum samples and cultured HUVEC and HTMC cells was first isolated using a commercially available DNA extraction assay kit (Tiangen, Beijing, China) in accordance with the protocol provided by the manufacturer. The genotyping of the SNP in MALAT1 was evaluated using a TaqMan allelic discrimination assay in conjunction with an ABI 7900 real‐time PCR machine (ABI, Foster City, CA) following the standard procedure recommended in the instruction manual comes with the assay kit.

### 2.4 RNA isolation and real‐time PCR

Total RNA in collected serum samples and cultured HUVEC and HTMC cells was first isolated using the Trizol agent (Invitrogen, Carlsbad, CA, USA) following the standard procedure recommended in the instruction manual come with the assay kit. The level of MALAT and miR‐1 expression in different samples was evaluated by a One‐Step SYBR RT‐RNA PCR assay kit (TaKaRa, Tokyo, Japan) following the standard procedure recommended in the instruction manual come with the assay kit. After the operation of real‐time PCR, the levels of relative MALAT (Forward primer: 5′‐GAAGATAGGCATTTGAGTGGCT‐3′; Reverse primer: 5′‐CTGAAGAGCATTGGAGATCAGC‐3′) and miR‐1 (Forward primer: 5′‐TGGAATGTAAAGAAGTATGT‐3′; Reverse primer: 5′‐GAACATGTCTGCGTATCTC‐3′) expression were quantified using the expression of housekeeping gene GAPDH (Forward primer: 5′‐GCACCGTCAAGGCTGAGAAC‐3′; Reverse primer: 5′‐GCCTTCTCCATGGTGGTGAA‐3′) and U6 (Forward primer: 5′‐CTCGCTTCGGCAGCACAT‐3′; Reverse primer: 5′‐TTTGCGTGTCATCCTTGCG‐3′) as the internal reference. The calculation of MALAT and miR‐1 expression was based on the conventional 2^−ΔΔCt^ method.

### 2.5 Cell culture and cell transfection

HUVEC and HTMC cells were purchased from the American Type Culture Collection (ATCC, Manassas, VA, USA) and maintained in RPMI‐1640 medium containing appropriate antibiotics and 10% foetal bovine serum. All cell culture supplies were purchased from Invitrogen (Carlsbad, CA, USA). The cells were subsequently divided into two groups, that is an empty vector control group and a P‐MALAT1 group. The cells were then transfected with corresponding plasmids using Lipofectamine 3000 (Invitrogen) following the standard procedure recommended in the instruction manual come with the transfection reagent.

### 2.6 Vector construction, mutagenesis and luciferase assay

The fragment sequences of miR‐1 and IL‐6 3’ UTR containing the potential binding sites of MALAT1 and miR‐1, respectively, were inserted into separate pcDNA vectors (Promega, Madison, WI, USA) to generate the wide‐type plasmids for miR‐1 and IL‐6 3’ UTR, respectively. Then, site‐directed mutagenesis was carried out in the MALAT1 and miR‐1 binding sites, respectively, located on the miR‐1 and IL‐6 3′ UTR using a Quick Change 5 mutagenesis kit (Stratagene, San Diego, CA, USA) to produce the mutant fragment sequences of miR‐1 and IL‐6 3′ UTR, respectively, which were subsequently inserted into separate pcDNA vectors to generate the mutant type plasmids for miR‐1 and IL‐6 3′ UTR, respectively. Then, the vectors (wild‐type or mutant miR‐1 or IL‐6) were cotransfected into HUVEC and HTMC cells in conjunction with MALAT1 or miR‐1 mimics using Lipofectamine 3000 (Invitrogen) following the standard procedure. At 48 hours after the start of transfection, the luciferase activity of transfected cells was measured by a dual‐luciferase reporter assay system (Promega Corporation) and the endogenous control was *Renilla* luciferase plasmid.

### 2.7 ELISA

The serum expression of IL‐6 in blood samples collected from subjects in various groups of patients was quantified using a commercial ELISA kit for IL‐6 (Bio‐rad Laboratory, Hercules, CA, USA) following the standard procedure recommended in the instruction manual come with the transfection reagent.

### 2.8 Western blot

The protein expression of IL‐6 in collected serum samples and cultured HUVEC and HTMC cells was evaluated using Western blot. First, the protein content in each sample was isolated by lysing the sample using a RIPA buffer (Beyotime, Shanghai, China). Then, the protein samples were resolved by 10% SDS‐PAGE and blotted onto PVDF membranes, which were then blocked using 5% skim milk, incubated with primary anti‐IL‐6 antibodies (dilution 1:2000, ab6672, Abcam, Cambridge, MA, USA) and secondary antibodies labelled with horseradish peroxidase (dilution 1:10 000, ab6721, Abcam, Cambridge, MA, USA) in sequence using the conventional procedure and developed using a Bio‐Rad ChemiDoc imaging system (Bio‐rad Laboratory, Hercules, CA, USA). Finally, the relative expression of IL‐6 proteins in the samples was quantified using Image Lab software (Bio‐rad Laboratory) and β‐actin as internal control.

### 2.9 Statistical analysis

SPSS 20.0 for Windows (IBM, Chicago, IL, USA) was utilized to carry out all statistical analyses. All categorical data were displayed in numbers (percentages) and were evaluated using the chi‐square tests. All continuous variables were evaluated using Student's *t* tests and displayed in mean ± standard deviations. The distribution of different genotypes of MALAT1 SNP and its correlation with the risk of NTG were assessed with the Multivariate logistic regression. The *P*‐value of inter‐group comparisons was calculated using independent *t* test (comparison between two groups) or one‐way ANOVA (comparison between three or more groups). The analyses of haplotypes and linkage disequilibrium were carried out using SHEsis software (http://analysis.bio‐x.cn/myAnalysis.php). Multivariate logistic regression was carried out for independent variables, including age, gender, history of systemic diseases such as hypertension, diabetes mellitus and hyperlipidaemia, and body mass index. A *P*‐value less than 0.05 of the difference during data analysis was considered as significant. Each experiment of this study was performed in triplicate.

## 3 RESULTS

### 3.1 The characteristics of patients

Basic information of 402 NTG patients and 425 healthy participants were collected and summarized in Table 1. Student's *t* test was utilized to perform the statistical comparison, and the results revealed no obvious difference in all patient characteristics between the above two groups.

**Table 1:** Demographic parameters of the participant of this study

Characteristics	Control (N = 425)	NTG (N = 402)	*P*‐value
Sex, male	254 (59.8)	226 (56.2)	0.573
Age, years	64.5 ± 5.1	63.8 ± 6.5	0.614
Systemic diseases (n, %)			
Hypertension	379 (50.3)	375 (49.7)	0.625
Diabetes mellitus	263 (47.9)	286 (52.1)	0.542
Hyperlipidaemia	255 (48.7)	269 (51.3)	0.716
Body mass index, kg/m2	25.8 ± 7.2	25.5 ± 6.7	0.785

### 3.2 Association between NTG risk and MALAT1 polymorphisms and haplotype

The distributions of the four polymorphisms (rs619586, rs11227209, rs664589 and rs3200401) in lncRNA MALAT1 of NTG patients and controls were listed in Table 2. As shown in Table 2, genotype and allele frequencies of four polymorphisms of lncRNA MALAT1 showed no significant association with the risk of NTG (*P* > 0.05). LD (Linkage disequilibrium) analysis showed a moderate or strong LD among the above four polymorphisms in lncRNA MALAT1. As shown in Table 3, six common haplotypes were detected: CACC, GGGT, CGCC, CAGC, GAGT and CACT. Compared to the other haplotypes, the GGGT haplotype was associated with a decreased risk of NTG (*P* < 0.001), while other five haplotypes were not associated with the risk of NTG (*P* > 0.05).

**Table 2:** Genotype and allele comparison between control and NTG groups

Characteristics	Control (N = 425)	NTG (N = 402)	Adjusted OR (95% CI)	*P*‐value
rs11227209				
CC	338 (79.5)	311 (79.5)	1.00	
CG	79 (18.6)	79 (19.7)	1.05 (0.73‐1.54)	0.84
CG/GG	87 (20.5)	91 (22.6)	1.01 (0.71‐1.44)	0.91
C allele	755 (88.8)	701 (87.2)	1.00	
G allele	95 (11.2)	103 (12.8)	1.03 (0.72‐1.38)	0.92
rs619586				
AA	312 (73.4)	301 (74.9)	1.00	
AG	82 (19.3)	77 (19.2)	1.04 (0.83‐1.43)	0.81
AG/GG	113 (26.6)	101 (25.1)	1.02 (0.97‐1.34)	0.94
C allele	706 (83.1)	679 (84.5)	1.00	
G allele	144 (16.9)	125 (15.5)	1.05 (0.79‐1.48)	0.95
rs664589				
CC	308 (72.5)	299 (74.4)	1.00	
CG	93 (21.9)	81 (20.1)	1.01 (0.87‐1.53)	0.74
CG/GG	117 (27.5)	103 (25.6)	0.96 (0.83‐1.64)	0.78
C allele	709 (83.4)	679 (84.5)	1.00	
G allele	141 (16.6)	125 (15.5)	1.01 (0.87‐1.39)	0.91
rs3200401				
CC	305 (71.8)	295 (73.4)	1.00	
CT	98 (23.1)	88 (21.9)	1.02 (0.85‐1.37)	0.68
CT/TT	120 (28.2)	107 (26.6)	1.04 (0.91‐1.48)	0.79
C allele	708 (83.3)	678 (84.3)	1.00	
T allele	142 (16.7)	126 (15.7)	1.01 (0.78‐1.33)	0.89

**Table 3:** Haplotype analysis between control and NTG groups

Haplotypes	Control (N = 425)	NTG (N = 402)	Adjusted OR (95% CI)	*P*‐value
CACC	327 (76.9)	321 (79.9)	1.00	
GGGT	43 (10.1)	31 (7.7)	0.79 (0.53‐1.16)	0.84
CGCC	28 (6.6)	6 (1.5)	0.31 (0.14‐0.47)	<0.001
CAGC	9 (2.1)	13 (3.2)	1.11 (0.72‐1.69)	0.78
GAGT	7 (1.6)	15 (3.7)	1.36 (0.71‐1.88)	0.92
CACT	11 (2.6)	16 (4.0)	1.41 (0.68‐1.93)	0.59

### 3.3 MALAT1 haplotypes were correlated with the expression of MALAT1, miR‐1 and IL‐6

**Figure 1:**
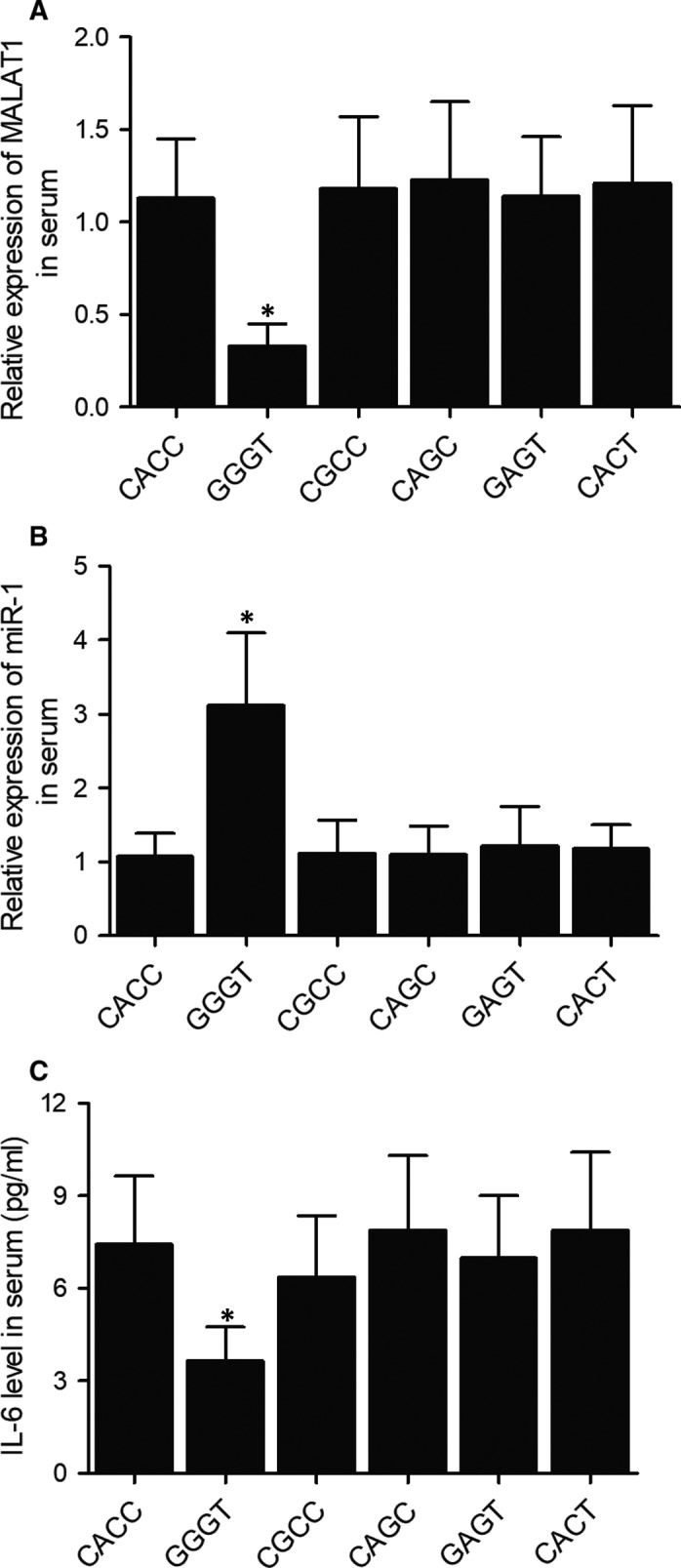
NTG patients carrying different MALAT1 haplotypes showed distinct MALAT1, miR‐1 and IL‐6 expression. A, MALAT1 expression in serum was diminished in patients carrying the GGGT haplotype (*P‐value < 0.05 compared with CACC group). B, miR‐1 expression in serum was increased in patients carrying the GGGT haplotype (*P‐value < 0.05 compared with CACC group). C, IL‐6 in serum was decreased in patients carrying the GGGT haplotype (*P‐value < 0.05 compared with CACC group)

402 NTG patients were enrolled in this study and divided into 6 groups according to their haplotypes of MALAT1 (CACC, GGGT, CGCC, CAGC, GAGT and CACT). The serum samples of all NTG patients with different haplotypes of MALAT1 were collected to evaluate their expression of MALAT1, miR‐1 and IL‐6. As shown in Figure 1, the expression of MALAT1 was significantly decreased in NTG patients carrying the GGGT haplotype (Figure 1A). On the contrary, the expression of serum miR‐1 was remarkably increased in NTG patients carrying the GGGT haplotype (Figure 1B), while serum IL‐6 expression was notably diminished in NTG patients carrying the GGGT haplotype (Figure 1C).

### 3.4 Different RNFL thickness, RA and C/D ratio in NTG patients carrying different MALAT1 haplotypes

**Figure 2:**
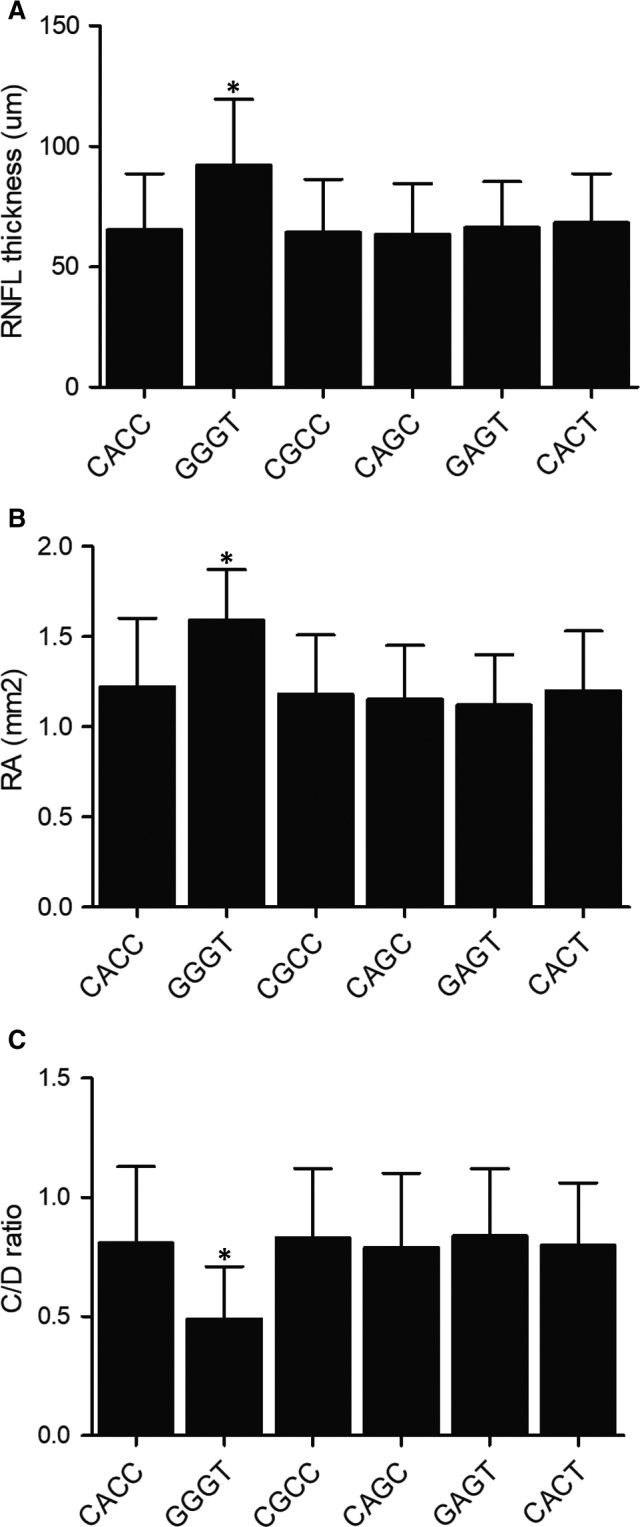
Different RNFL thickness, RA and C/D ratio were observed in NTG patients carrying the different MALAT1 haplotypes. A, RNFL thickness was increased in patients carrying the GGGT haplotype (*P‐value < 0.05 compared with CACC group). B, RA was larger in patients carrying the GGGT haplotype (*P‐value < 0.05 compared with CACC group). C, C/D ratio was reduced in patients carrying the GGGT haplotype (*P‐value < 0.05 compared with CACC group)

RNFL thickness was measured in NTG patients carrying different haplotypes of MALAT1 (CACC, GGGT, CGCC, CAGC, GAGT and CACT). The NTG patients carrying the GGGT haplotype had a thicker RNFL (retinal nerve fibre layer) than patients carrying other MALAT1 haplotypes (Figure 2A), and the RA (rim area) was also larger in patients carrying the GGGT haplotype (Figure 2B). Furthermore, the C/D ratio in patients carrying the GGGT haplotype was significantly reduced when compared with those carrying the CACC, CGCC, CAGC, GAGT and CACT haplotypes (Figure 2C).

### 3.5 Mechanistic study unveiling the regulatory network of miR‐1, MALAT1 and IL‐6

**Figure 3:**
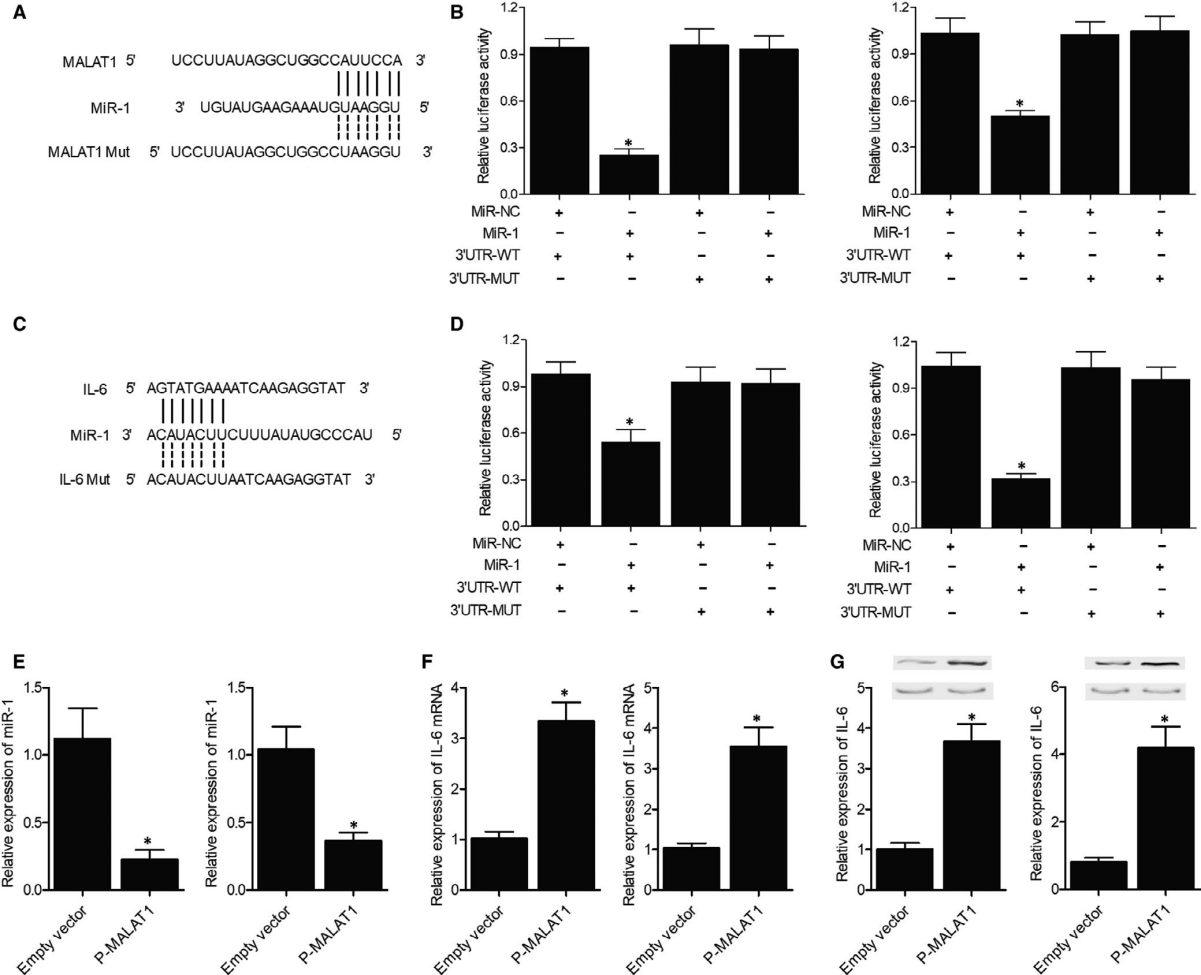
MALAT1 inhibited miR‐1 and increased IL‐6 expression. A, Sequence analysis showed a potential binding between miR‐1 to MALAT1. B, Luciferase assay revealed inhibition of MALAT1 expression by miR‐1 (*P‐value < 0.05 compared with miRNA control group). C, Sequence analysis showed potential binding between miR‐1 and IL‐6. D, Luciferase assay revealed inhibition of IL‐6 expression by miR‐1 (*P‐value < 0.05 compared with miRNA control group). E, miR‐1 expression was suppressed by transfection of MALAT1 in HUVEC and HTMC cells (*p‐value < 0.05 compared with empty vector group). F, IL‐6 mRNA expression was promoted in HUVEC and HTMC cells transfected with MALAT1 (*P‐value < 0.05 compared with empty vector group). G, IL‐6 protein expression was elevated in HUVEC and HTMC cells transfected with MALAT1 (*P‐value < 0.05 compared with empty vector group)

Sequence analysis indicated that a binding site of miR‐1 existed on MALAT1 (Figure 3A). Luciferase assay was performed in HUVEC and HTMC cells using vectors carrying WT and mutant sequences of MALAT1 promoter containing the miR‐1 binding site. MiR‐1 inhibited the luciferase activity of the WT vector but not that of the mutant vector in both HUVEC and HTMC cells, suggesting an inhibitory role of miR‐1 in MALAT1 expression (Figure 3B). Moreover, a binding site of miR‐1 was also found on the 3’ UTR of IL‐6 (Figure 3C). Luciferase assay in HUVEC and HTMC cells also showed that miR‐1 directly suppressed the expression of IL‐6 by binding to the 3’ UTR of IL‐6 (Figure 3D).

After we transfected MALAT1 into HUVEC and HTMC cells, quantitative real‐time PCR was carried out to check the expression of miR‐1 and IL‐6 mRNA. The expression of miR‐1 was apparently reduced in HUVEC and HTMC cells transfected with MALAT1 (Figure 3E). Accordingly, the expression of IL‐6 in both types of cells was remarkably enhanced after transfection with MALAT1 (Figure 3F). Western blot was used to evaluate the expression of IL‐6 protein after transfection with MALAT1. The results clearly confirmed that the protein expression of IL‐6 in HUVEC and HTMC cells was evidently promoted by the transfection with MALAT1 (Figure 3G).

## 4 DISCUSSION

MALAT1 can contribute to tumorigenesis.^32^ MALAT1 also regulates immune reactions via interacting with NF‐κB.^33^ MALAT1 was also deregulated in patients with MS.^34^ It was demonstrated that the protective role of MALAT1 in glaucoma patients is played via the PI3K/Akt cascade to reduce the apoptosis of RGCs.^26^

Shi et al (2013) showed that interactions among IL‐6 rs2069837 SNP, age of primiparity, as well as menopausal status can increase the risk of cervical cancer.^35^ Yang et al (2017) showed that lncRNA GAS5 can suppress cervical cancer by down‐regulating miR‐196a as well as miR‐205 expression.^36^ In their study, the relationship between precancerous cervical lesions and SNPs in the lncRNA MALAT1 and THRIL was studied to show that AG genotype of rs7133268 in THRIL is involved in the decreased susceptibility to precancerous cervical lesions. In this study, we enrolled NTG patients and divided them into groups according to the differential haplotypes of MALAT1. We found that genotype and allele frequencies of four polymorphisms in lncRNA MALAT1 had no significant association with NTG risk, while GGGT haplotype was associated with a decreased risk of NTG. In addition, we carried out qPCR to evaluate the expression of MALAT1 and miR‐1 in the serum of NTG patients carrying different haplotypes of MALAT1. The MALAT1 expression was obviously down‐regulated in NTG patients carrying the GGGT haplotype, along with up‐regulated expression of miR‐1.

MiR‐1 was shown to have a great potential in blocking cancer progression as well as in reducing resistance to chemotherapy in many malignancies, including lung cancer as well as colorectal cancer.^37^ Moreover, miR‐1 level is reduced in tissues of prostate cancer patients and in mice xenografted with PC cells.^38^, ^39^, ^40^ Furthermore, decreased miR‐1 level is positively related to a poor outcome of PC patients.^39^ It was shown that miR‐1 is reduced in tissues of breast cancer patients with a lower rate of survival.^41^ In this study, we performed luciferase assay to explore the regulatory correlation among MALAT1, miR‐1 and IL‐6. Both MALAT1 and IL‐6 were targets of miR‐1, indicating that miR‐1 plays an inhibitory role in MALAT1 and IL‐6 expression. Meanwhile, we transfected MALAT1 into HUVEC and HTMC cells and checked the effect of MALAT1 on miR‐1 and IL‐6 expression. MiR‐1 was evidently down‐regulated while IL‐6 was up‐regulated by MALAT1.

As a soluble protein, IL‐6 can mediate inflammation, immune reactions, as well as hematopoiesis.^4^ In humans, IL‐6 contains about 212 amino acids, which are distributed in different components such as a signalling peptide containing 28 amino acids as well as a 20 kD core protein. Previously, the serum expression of IL‐6 was shown to be higher in patients with NTG, which was consistent with the results obtained by Huang et al studying the level of serum IL‐6 expression in patients with primary open‐angle glaucoma.^42^, ^43^ In addition, the G allele in the (−174) SNP of IL‐6 was demonstrated to elevate the expression of IL‐6 proteins.^11^ This correlation between the SNP of IL‐6 and the pathogenesis of glaucoma still remains unclear, although it was shown to be involved in the pathogenesis of Alzheimer's disease.^44^ A past study showed that the serum expression of IL‐6 was higher in patients with NTG, which was inconsistent with the results obtained by Gani et al.^45^, ^46^ Moreover, the change in serum levels of IL‐6 is involved in the progression of glaucoma, indicating that disordered immune reactions may trigger the glaucomatous neuropathy in patients with NTG. Overall, existing data support the hypothesis that the presence of inflammation in neuron cells accelerates the development of NTG. In this study, we performed ELISA to analyse the expression of IL‐6 in the serum of patients carrying different haplotypes of MALAT1 and found that IL‐6 was dramatically decreased in patients carrying the GGGT haplotype. Furthermore, we analysed the RNFL thickness, RA and C/D ratio of NTG patients carrying differential haplotypes of MALAT1. RNFL thickness and RA was notably increased, while the C/D ratio was evidently declined in patients carrying the GGGT haplotype.

## 5 CONCLUSION

We concluded that the MALAT1 GGGT haplotype is associated with the prognosis of NTG patients. The MALAT1 polymorphism and serum IL‐6 levels may be associated with the severity of NTG. Further genetic studies of NTG are necessary to investigate the genetic basis and factors involved in the development of NTG.

## CONFLICT OF INTEREST

The authors declare that they have no competing interests.

## AUTHOR CONTRIBUTION

**Jinliang Yue:** Conceptualization (equal); Investigation (equal); Methodology (equal); Software (equal). **Shu‐feng Zheng:** Investigation (equal); Supervision (equal); Writing‐original draft (equal); Writing‐review & editing (equal).

## ETHICAL APPROVAL AND CONSENT TO PARTICIPATE

The study was approved by the institutional ethics committee and written consent forms were obtained before the initiation of this study.

## Data Availability

The data that support the findings of this study are available from the corresponding author upon reasonable request.
